# The Association of the *BRAF*-V600E Mutation with the Expression of the Molecular Markers in the Primary Tumor and Metastatic Tissue in Papillary Thyroid Cancer

**DOI:** 10.31557/APJCP.2021.22.7.2017

**Published:** 2021-07

**Authors:** Liudmila V. Spirina, Svetlana Yu. Chizhevskaya, Irina V. Kovaleva, Irina V. Kondakova

**Affiliations:** 1 *Laboratory of Tumor Biochemistry, Cancer Research Institute, Tomsk National Research Medical Center, Russian Academy of Medical Sciences, Russian Federation. *; 2 *Division of Biochemistry and Molecular, Russian Federation. *; 3 *Division of Head and Neck Cancers, Cancer Research Institute, Tomsk National Research Medical Center, Russian Academy of Medical Sciences, Russian Federation. *; 4 *Division of Oncology, Siberian State Medical University, Russian Federation. *; 5 *Siberian State Medical University, Tomsk, Russian Federation. *; 6 *Cancer Research Institute, Siberian State Medical University, Tomsk, Russian Federation. *; 7 *Cancer Research Institute, Tomsk National Research Medical Center, Russian Academy of Sciences, Tomsk, Russian Federation. *

**Keywords:** Thyroid cancer, BRAF-V600E, primary tumor, metastases, molecular factors

## Abstract

**Objective:**

The aim of this study was investigation the AKT / mTOR signaling pathway components, transcriptional and growth factors, as well as steroid hormone receptors and nuclear factors Brn-3α and TRIM16 expression in the tissue of the primary thyroid tumor and metastases, depending on the *BRAF*- V600E status.

**Material and Methods:**

The study was enrolled 20 patients with PTCs, who underwent surgical treatment. They were divided into negative *BRAF*-V600E status (12 people), positive *BRAF*-V600E status (8 patients). Mutation status was assessed in paired metastatic tissue samples. The molecular marker expression was determined by real-time PCR. The Real-time-PCR-*BRAF*-V600E reagent kit evaluated the *BRAF*-V600E mutation.

**Results:**

A decrease in the PDK kinase, PTEN, VHL mRNA level in primary cancers was noted, compared with metastases’ tissue. An increase in AKT, GSK-3β, mTOR, 70s 6 kinase was revealed in cancers with point mutation compared with the primary tumor without a mutation. Positive mutation status was accompanied by an increase in NF-κB p65, NF-κBp50, VEGF HIF-2 VHL level compared to the primary tumor with negative *BRAF*-V600E status. In the metastases with the *BRAF*-V600E point mutation, a decrease in the PDK kinase, HIF-1; VHL; TRIM16, and ERα expression was observed, compared to lymph node metastases (LNMs) without the mutation. The concordance in the *BRAF*-V600E tumor status and LNMs was observed only in 50% of patients. If the *BRAF* gene status did not match PTCs and LNMs, an increase in the mTOR, NFkBp65, VHL, and ERα mRNA levels was found in the PTCs. In LNMs, there was an increase in the c-RAF PTEN NFkBp65 VHL expression compared to non-concordant ones.

**Conclusion:**

The heterogeneity in the primary tissue’s expression profile and metastases was noted. The *BRAF*-V600E mutation can affect the molecular characteristics both in the primary cancers and metastases. The discrepancy between the mutant status and the molecular factors expression variability in the primary tumor and LNMs determines its progression.

## Introduction

Thyroid cancer is a dynamic disease; during disease, cancers generally become more heterogeneous (Albini et al., 2012). Heterogeneity provides the fuel for resistance; therefore, an accurate assessment of tumor heterogeneity is essential for developing effective therapies. Thyroid cancer is a heterogeneous group of diseases. The current molecular markers that have received the most attention in thyroid cancer include *BRAF*,*.*point mutations, and PAX8/PPARG and RET/PTC rearrangements (Bhagirath et al., 2019).

Despite the rapidly developing field of molecular markers, several limitations exist. Thyroid cancer behavior is defined by the effects of the initiating oncogene and secondary events in tumor cells and the tumor microenvironment that is genetic and epigenetic (Ge et al., 2020). The long-observed age-associated mortality risk in PTC is dependent on *BRAF* status; age is an intense, continuous, and independent mortality risk factor in patients with *BRAF*-V600E mutation but not in patients with wild-type *BRAF*. These results question the conventional general use of patient age as a high-risk factor in PTC and call for differentiation between patients with *BRAF*-V600E and wild-type *BRAF* when applying age to risk stratification and management of PTC (Califano et al., 2018).

The *BRAF*-V600E point mutation is the most common genetic mutation detected in patients with papillary thyroid cancer (PTC) and occurs in approximately 45–60% of patients (Califano et al., 2018; Chen et al., 2018). Over the past several years, there has been intense focus on identifying molecular markers to predict the aggressiveness of thyroid cancers better and define therapeutic targets. It is known that the c-RAF protein’s mutation with the formation of the b-RAF oncoprotein has a significant effect on cancer progression. Patients with PTC have biological markers associated with this somatic mutation. According to the data presented in previous work, the *BRAF*-V600E mutation is accompanied by an increase in the growth and transcription factors mRNA level with a reduction in the AKT/mTOR signaling pathway activity (Chen et al., 2020). 

The presented molecular parameters are responsible for angiogenesis, the immune response initiation, and apoptosis blockage resulting in abnormal cell growth with the potential to invade or spread to other parts of the body (Albini et al., 2012). Angiogenesis also plays a crucial role in spreading cancer cells and colonization at the body’s distant locations and metastasis formation (Dagogo-Jack et al., 2018). It is regulated by hypoxia-inducible factors and, followed by the elevated VEGF, is responsible for the vessel formation. The von Hippel-Lindau (VHL) tumor suppressor gene’s significance in the PTC progression seems to be essential (Gawin et al., 2019). The lower VHL expression in cancers is a sign of the aggressive tumor’s behavior and neoangiogenesis initiation (Halon et al., 2012). Angiogenesis is a key event related to tumor progression in PTC (Jena et al., 2019). 

Recently, the system’s importance for controlling hormone receptor expression mediated by nuclear peptides became evident in PTC. The transcription factor Brn-3α can affect androgen and estrogen receptors (Kurtulmus et al., 2016; Lodewijk ey al., 2017). 

It is observed the participation of Tripartite motif 16 (TRIM16) protein in the pathogenesis of hormone-dependent tumors due to its “anti-estrogenic effect” (Tan et al., 2017). The effect of TRIM16 on epithelial-mesenchymal transition (EMT) and metastasis in cancer cells has a clinical significance in cancer progression (Melo et al., 2017). The PTC is characterized by an increased level ERβ, AR mRNA, which is related to the peculiarities in the nuclear factors Brn-3a, TRIM16 level (Magri et al., 2012). Nuclear factors Brn-3α and TRIM16 modulating expression of steroid hormones play an essential role in developing thyroid tumors. It was found TRIM16 mRNA level was associated with the expression of ERβ, which seems to be mediated by its “anti-estrogen effect” (Spirina et al., 2018; Jena et al., 2019). 

Little is known about the frequency of critical mutations in thyroid cancer metastases and their relationship with the primary tumor genotype. The distant metastasis in PTC is associated with an adverse disease outcome. It is widely known that an unfavorable outcome characterizes the development of metastases. In half of the cases, lymph node metastases (LNMs) were found in patients undergoing surgical thyroid resection (Lu et al, 2015; Parameswaran R, et al, 2017).

*BRAF*, RAS, and TERT mutations are highly prevalent in metastatic differentiated thyroid cancer and are concordant between primary and metastatic cancers (Sadow et al., 2010). The value of selected mutations for tumor risk stratification and assessment of patients’ prognosis was affirmed. To date, there are ideas about its importance in the aggressive tumor potential in oncogenesis, cancer behavior, and patient’s outcome (Spirina et al., 2018; Spirina et al., 2019). The high concordance for all the genes was found comparing the genotype of primary tumors with lymph node metastasis (LNMs). On the other hand, distant metastases show enrichment in TERTp mutations and decrease *BRAF* mutations (Sadow et al., 2010).

Currently, the heterogeneity of the *BRAF*-V600E status of the primary tumor and metastases, concordance, and tissue coincidence are widely studied. It is noted that the coincidence of the gene status is observed in the majority of patients (95.2%) (Sohn et al., 2016). Only 4.8% had a heterogeneous status for this mutation, which was observed in patients with a disease’s recurrent course. In turn, there is information about the protective effect of the *BRAF*-V600E mutation and a decrease in the likelihood of lung damage in papillary thyroid cancer (Shen et al., 2018). Despite the previous data of unfavorable prognosis in mutant b-RAF protein turnover, it is obtained to be a sign of response to radioiodine therapy in PTC (Califano et al., 2018). In common, Chen found (2020) no relationship between the *BRAF*-V600E mutation in LNM and its invasive characteristics in PTC (Chen et al., 2020).

Consequently, a tumor’s biological behavior is mediated by a complex of molecular markers, which include the AKT/mTOR signaling pathway components, transcription and growth factors, and steroid hormone receptors and nuclear factors associated with them. The study aimed to study the AKT / mTOR signaling pathway components, transcriptional and growth factors, as well as steroid hormone receptors and nuclear factors Brn-3α and TRIM16 expression in the tissue of the primary thyroid tumor and metastases, depending on the *BRAF*- V600E status. 

## Material and Methods

The study included 20 patients with PTC, T1-4N0-2M0 stages, who underwent surgical treatment in the Cancer Research Institute’s clinics, Tomsk National Research Medical Center. Patients were divided into two groups: without the *BRAF*-V600E mutation, which included 12 people, and with its presence - 8 people. Mutation status was assessed in paired metastatic tissue samples. We revealed in 6 metastases positive mutation status, and 14 ones had a negative status.

The study’s material was samples of the tumor, non-transformed tissue of the thyroid gland, located at least 1 cm from the tumor’s border, and tissue of affected and unaffected lymph nodes were frozen after collection and stored at t-80°C. The Local Committee approved the study for Medical Ethics, and all patients provided written informed consent. All procedures involving patients were carried out by the Declaration of Helsinki’s Declaration on Human Rights (1964). All patients signed informed consent to participate in the study.

*Isolation of DNA*

DNA was isolated using the FFPET DNA - Extraction Kit (Biolink, Russia). Its concentration was assessed on a NanoDrop-2000 spectrophotometer (Thermo Scientific, USA). The resulting DNA was used for real-time PCR. Determination of the *BRAF*-V600E mutation. The *BRAF*-V600E mutation was determined using the Real-time-PCR-*BRAF*-V600E reagent kit, designed to detect GTG GGG’s point mutation in codon 600 of the *BRAF* gene. The analysis is carried out by allele-specific real-time PCR.

*RNA extraction*

The postoperative tumor samples were incubated in RNAlater solution (Ambion, USA) for 24-hours at + 4 ^o^C and then stored at -80^o^C. Total RNA was extracted using RNeasy Mini Kit (Qiagen). 

*RT-qPCR*

PCR was conducted in 25 μl reaction volumes containing 12.5 μl BioMaster HS-qPCR SYBR Blue (2X) (“Biolabmix” Russia) and 300 nano M of each primer. Primers were selected using the Vector NTI Advance 11.5 software and the NCBI database (HTTP: //www.ncbi.nlm.nih.gov/nuccore) (Table 1).

A pre-incubation at 95°C for 10 min was to activate the Hot Start DNA polymerase and denature DNA. It was followed by 45 amplification cycles of 95°C denaturations at 95°C for 10 sec, 60°C annealings at 60°C for 20 sec (iCycler iQ™, BioRad).

The housekeeping gene of the GAPDH (glyceraldehyde-3-phosphate dehydrogenase) enzyme was used as a reference gene, and the expression level of each target gene was normalized to the expression of GAPDH. The fold changes were calculated by the ΔΔCt method (the total ΔΔCt = fold of cancerous/normal tissue gene level), using normal tissue. A ratio of specific mRNA/GADPH (GADPH as a respective control) amplification was then calculated.

The statistical analysis was performed using the Statistica 8.0 software package. Verification of normality was performed using the Kolmogorov-Smirnov test. The results of the determination of gene expression are presented as Me (Q1; Q3). The Mann-Whitney test assessed the significance of differences. Differences were considered significant at p <0.05. The significance of differences in the frequencies of qualitative traits was evaluated using the *χ*^2^ criterion with the Yates correction.

## Results

Molecular markers play a significant role in the oncogenesis and PTC spreading. Table 2 presents data on the AKT/mTOR signaling pathway components in the primary tumor and metastases. A decrease in the PDK kinase and PTEN phosphatase mRNA level was noted by 2.23 and 5.6 times in the metastases, respectively, compared with the primary tumor tissues. Simultaneously, the NF-κB p50 and VHL expression decreased by 32.4 and 14.8 times, respectively, in the metastases compared with the primary papillary tumors.

The detection of molecular alterations in thyroid carcinogenesis in the signaling pathways relates to some diagnostic, prognostic, and treatment issues. The clinical implications of *BRAF* altered gene and molecules impact the change in AKT/mTOR signaling pathway components, transcription and growth factors, hormone receptors, and nuclear factors Brn-3α, TRIM16 (Table 3). Mutation status in the primary tumor led to an increase in the AKT, GSK-3β, mTOR, the 70s 6 kinase expression in 19.9; 47.1; 164.3; 101.6 times, respectively with the primary tumor without a mutation. NF-κBp 65, NF-κBp50, VEGF, HIF-2; VHL elevated mRNA level by 10.6; 13.5; 240.6; 11.5; 368.5 times found in tumors with mutation, respectively, compared to the primary tumor with the wild variant of the *BRAF* gene.

On the contrary, positive *BRAF*-V600E status in the LNMs was associated with decreased molecular markers’ expression. PDK kinase; HIF-1; VHL; transcription factor TRIM16, and ERα mRNA level in metastases with a *BRAF*-V600E mutation was reduced in 2.1; 27, 42.33; 4.0; 42.4 and 8.0 times, respectively, compared to LNMs without this mutation.

*BRAF* mutations are associated with the abnormal regulation of microRNA, constitute the most frequent molecular alterations identified in PTC, determining cancer’s biological characteristics and the disease’s outcome. The heterogeneity in the primary tumor and LNMs is widely known, followed by the molecular and genetic markers’ change. The study revealed that the primary tumor mutation was observed in 40% of patients, while only 10% of patients retained it in the metastatic tissues (Table 4). Among patients with a negative status for the *BRAF*-V600E mutation (60% of the total number of patients), the same metastatic status was observed in 20% of patients. In 40% of patients, the mutation appeared in the LNMs.

Consequently, concordance in *BRAF*-V600E tumor status and metastases was observed only in 50% of patients: 10% for a positive status in the primary tumor and metastases and 40% for a negative status. The majority of patients with a positive tumor status in metastases did not have a mutation (30%). Only in 20% of patients from 60%, we found the *BRAF*-V600E point mutation in the LNMs in patients with a *BRAF*-V600E negative status in the primary tumors.

The molecular and genetic markers overlap are currently intensively studied. In the absence of concordance, an increase in the studied molecular markers expression was indicated in the primary tumor and metastases. If the *BRAF* gene status did not match in the cancers and metastases, an increase in the level of mTOR mRNA, NFkBp65; VHL; ERα was observed in 32.8; 67.3; 56.0; 1.5 times. In the LNMs with non-identical *BRAF* gene status in cancerous tissues, there was an increase in the c-RAF PTEN NFkBp65 VHL expression in 16.0; 13.3; 60.0; 36.9 times, respectively, compared to concordant tissues.

**Table 1:** Sequences of primers

Gene	Amplicon	Sequence
*CAIX* NM_001216.2	217 bp	F 5′-GTTGCTGTCTCGCTTGGAA-3′ R 5′-CAGGGTGTCAGAGAGGGTGT-3′
*HIF-1α* NM_001243084.1	188 bp	F 5′- CAAGAACCTACTGCTAATGCCA-3′ R 5′- TTTGGTGAGGCTGTCCGA-3′
*EPAS1* NM_001430.4	265 bp	F 5′- TGGAGTATGAAGAGCAAGCCT-3′ R 5′-GGGAACCTGCTCTTGCTGT-3′
*NFKB1* NM_001165412.1	144 bp	F 5′-CGTGTAAACCAAAGCCCTAAA-3′ R 5′-AACCAAGAAAGGAAGCCAAGT-3′
*RELA* NM_001145138.1	271 bp	F 5′-GGAGCACAGATACCACCAAGA-3′ R 5′-GGGTTGTTGTTGGTCTGGAT-3′
*PTEN* NM_001304717.2	136 bp	F 5′-GGGAATGGAGGGAATGCT-3′ R 5′-CGCAAACAACAAGCAGTGA-3′
*VEGFA* NM_001025366.2	316 bp	F 5′-AGGGCAGAATCATCACGAA-3′ R 5′-TCTTGCTCTATCTTTCTTTGGTCT-3′
*KDR* NM_002253.2	306 bp	F 5′-AACACAGCAGGAATCAGTCA-3′ R 5′-GTGGTGTCTGTGTCATCGGA-3′
*4EBP1* NM_004095.3	244 bp	F 5′- CAGCCCTTTCTCCCTCACT -3′ R 5′- TTCCCAAGCACATCAACCT -3′
*AKT1* NM_001014431.1	181 bp	F 5′- CGAGGACGCCAAGGAGA -3′ R 5′- GTCATCTTGGTCAGGTGGTGT -3′
*С-RAF* NM_002880.3	152 bp	F 5′- TGGTGTGTCCTGCTCCCT -3′ R 5′- ACTGCCTGCTACCTTACTTCCT -3′
*GSK3b* NM_001146156.1	267 bp	F 5′- AGACAAGGACGGCAGCAA -3′ R 5′-CTGGAGTAGAAGAAATAACGCAAT-3′
*70S kinase alpha* NM_001272042.1	244 bp	F 5′- CAGCACAGCAAATCCTCAGA -3′ R 5′- ACACATCTCCCTCTCCACCTT -3′
*m-TOR * NM_004958.3	160 bp	F 5′- CCAAAGGCAACAAGCGAT-3′ R 5′- TTCACCAAACCGTCTCCAA -3′
*PDK1* NM_001278549.1	187 bp	F 5′- TCACCAGGACAGCCAATACA -3′ R 5′- CTCCTCGGTCACTCATCTTCA -3′
*POU4F1* NM_006237	294 bp	F 5′- CACGCTCTCGCACAACAA-3′ R 5′- ATCCGCTTCTGCTTCTGTCT-3′
*AR* NM_000044	190 bp	F 5′- GAGGGACAGCAGGCAGA-3′ R 5′- GCTATCAGAACACACACACACACT-3′
*ESR1 ERα* NM_000125	386 bp	F 5′- TCCTGATGATTGGTCTCGTCT-3′ R 5′- GATGTGGGAGAGGATGAGGA-3′
*ESR2 ERβ* NM_001040275.1	243 bp	F 5′- GGTCCATCGCCAGTTATCAC-3′ R 5′- GCCTTACATCCTTCACACGA-3′
*TRIM16* NM_001348119	267 bp	F 5′- CAATGGAACGGGAAGGAG-3′ R 5′- GGACGGTGCTGGCTTCT-3′
*VHL * NM_000551.3	339 bp	F 5′- GGCAGGCGAATCTCTTGA-3′ R 5′- CTATTTCCTTTACTCAGCACCATT-3′
*GAPDH* NM_001256799.2	138 bp	F 5′- GGAAGTCAGGTGGAGCGA-3′ R 5′-GCAACAATATCCACTTTACCAGA-3′

Note: NM is the RNA sequence number in the NCBI Nucleotide Database (http://www.ncbi.nlm.nih.gov/nuccore); F, forward primer; R, reverse primer.

**Table 2:** Molecular Markers Expression in the Primary Tumor and Metastases in PTC

Indicator, Relative Units	Primary tumor	Metastases
AKT / mTOR signaling pathway components		
PDK	2,88 (1,00; 147,36)	1,26 (0,00;2,02)*
AKT	1,21 (0,38; 3,21)	0,73 (0,38; 1,81)
c-RAF	3,09 (0,38; 64,00)	0,31 (0,05;3,84)
GSK-3β	1,22 (0,39; 24,60)	3,81 (0,78;11,92)
mTOR	1,65 (0,07; 16,44)	2,15 (0,50; 4,10)
70s 6 киназа	1,19 (0,38; 11,65)	0,67 (0,27; 2,00)
4EBP1	0,12 (0,01; 0,74)	2,28 (0,00; 3,32)
PTEN	5,39 (0,50; 14,39))	0,96 (0,02; 1,30)*
Transcription and growth factors		
NFκBp65	27,58 (0,76; 64,38)	4,46 (0,13; 56,00)
NFκBp50	27,62 (3,72; 89,33)	0,85 (0,35;16,00)*
VEGFR2	0,44 (0,06; 8,59)	0,49 (0,10; 7,26)
VEGF	0,69 (0,13; 12,13)	1,25 (0,02;12,13)
CA9	2,13 (0,50;7,01)	0,37 (0,03;8,57)
HIF-2	1,13 (0,43; 3,03))	1,52 (0,13; 27,62)
HIF-1	5,35 (0,50; 16,62)	3,28 (0,12; 13,72)
VHL	20,1 ((0,85; 154,0)	1,36 (0,09; 5,59)*
Hormone reception		
TRIM16	0,13 (0,01; 1,73)	2,83 (0,18; 7,63)
Brn	1,54 (0,22; 21,56)	5,53 (0,38; 11,59)
AR	0,45 (0,02; 11,49)	0,54 (0,02; 2,00)
ERα	1,44 (1,02; 18,10)	1,52 (1,00; 8,00)
ERβ	1,09 (0,76; 6,70)	0,77 (0,10; 1,96)

*, the significance of differences in comparison with the primary tumor, p <0.05;

**Table 3:** Molecular Markers Expression in the Pprimary Tumor, Metastases and the *BRAF*-V600E Status

Indicator, Relative Unit	Primary tumor without mutation	Primary tumor with BRAF-V600E mutation	Metastases without mutation	Metastases with BRAF-V600E mutation
AKT / mTOR signaling pathway components				
PDK	1.26 (0.60; 2.24)	195.68 (104.37; 264.10)	2.02 (1.77; 113.71)	1.00 (0.01; 1.52)**
AKT	0.69 (0.06; 0.74)	13.78 (2.97; 152.19)*	0.78 (0.68; 1.02)	0.54 (0.38; 15.96)
c-RAF	0.89 (0.25; 64.0)	4.82 (3.09; 219.63)	2.74 (0.25; 3.84)	0.38 (0.04; 4.00)
GSK-3β	0.65 (0.02; 1.65)	30.62 (12.49; 40.62)*	11.92 (0.90; 23.35)	4.00 (3.63; 6.06)
mTOR	0.26 (0.01; 1.00)	42.72 (12.42; 361.00)*	4.10 (2.00; 79.00)	2.30 (0.50; 2.39)
70s 6 киназа	0.65 (0.25; 4.00)	66.06 (1.14; 141.26*)	1.17 (0.75; 24.55)	0.38 (0.27; 2.00)
4EBP1	0.22 (0.00; 0.74)	0.05 (0.02; 18.45)	2.57 (2.56; 3.80)	2.00 (0.00; 3.32)
PTEN	0.63 (0.24; 4.00)	31.19 (12.32; 54.95)	1.16 (0.15; 1.30)	0.76 (0.02; 2.00)
Transcription and growth factors				
NFκBp65	5.16 (0.50; 51.20)	54.59 (27.58; 95.19)*	1.16 (0.13; 7.16)	51.20 (1.76; 69.66)
NFκBp50	6.96 (0.50; 32.00)	93.09 (56.28; 120.93)*	16.00 (1.22; 150.15)	0.49 (0.38; 8.57)
VEGFR2	0.12 (0.06; 0.76)	40.52 (4.03; 92.82)	7.26 (0.23; 68.21)	0.76 (0.10; 6.06)
VEGF	0.36 (0.13; 1.00)	86.61 (0.98; 189.12)*	0.82 (0.03; 21.00)	6.06 (1.69; 12.13)
CA9	1.56 (0.19; 4.00)	17.20 (1.49; 46.98)	0.56 (0.08; 86.83)	0.96 (0.19; 8.57)
HIF-2	0.91 (0.25; 1.27)	10.48 (1.32; 82.87)*	1.54 (1.50; 27.62)	0.76 (0.09; 3.03)
HIF-1	0.50 (0.25; 16.62)	10.70 (5.35; 246.83)	13.72 (7.28; 46.44)	0.50 (0.12; 6.06)*
VHL	0.81 (0.27; 54.00)	36.85 (12.3;301.95)*	3.81 (1.70; 34.17)	0.09 (0.01; 0.12)**
Hormone reception				
TRIM16	0.47 (0.42;1.20)	0.05 (0.00; 35.13)	7.63 (4.55; 27.52)	0.18 (0.02; 6.80)*
Brn3α	0.22 (0.04; 0.65)	54.59 (13.92; 136.67)	3.06 (1.23; 99.22)	8.00 (0.38; 11.59)
AR	0.22 (0.01; 0.89)	59.43 (5.75; 364.26)	1.44 (0.57; 38.37)	0.19 (0.01; 2.00)
ERα	1.22 (0.44; 1.36)	24.95 (15.36; 80.75)	8.00 (1.53; 15.94)	1.00 (0.03; 1.52)**
ERβ	0.84 (0.42; 1.20)	11.68 (3.89; 35.13)	1.71 (0.52; 29.70)	0.76 (0.09; 0.78)

*, the significance of the differences in comparison with the primary tumor without mutation; p <0.05; **, the significance of the differences compared to the metastases without mutation, p <0.05

**Table 4:** *BRAF*-V600E Status in primary Cancers and Metastases in PTC

BRAF-V600E status in primary tumor	*BRAF*-V600E status in metastasis	Concordance of *BRAF*-V600E tumor status and metastases
8 patients – positive in *BRAF-*V600E - 40%	2 patients – positive in BRAF-V600E - 10% 6 patients - negative in BRAF-V600E - 30%	10%
12 patients – negative in *BRAF*-V600E - 60%	4 patients - positive in BRAF-V600E 20% 8 patients - negative in BRAF-V600E 40%	40%

**Table 5:** Molecular Markers Expression in Primary Tumor and Metastases Depending on the Coincidence of *BRAF*-V600E Status in the Primary Tumor and Metastatic Tissue

Indicator. Relative Unit	Primary tumor		Metastases	
	Primary tumor and metastasis have the same status *BRAF*-V600E	Primary tumor and metastasis have heterogenous *BRAF*-V600E status	Primary tumor and metastasis have the same status *BRAF*-V600E	Primary tumor and metastasis have heterogenous *BRAF*-V600E status
AKT / mTOR signaling pathway components				
PDK	2.24 (0.60; 3.52)	1.52 (1.00; 284.21)	1.77 (0.01; 2.02)	1.52 (1.00; 115.00)
AKT	1.00 (0.06; 1.43)	1.00 (0.38; 280.03)	0.78 (0.68; 1.02)	0.54 (0.38; 273.00)
c-RAF	6.24 (1.41; 64.00)	0.38 (0.25; 433.03)	0.25 (0.05; 2.74)	4.00 (0.38; 33.700)**
GSK-3β	0.80 (0.02; 1.65)	0.50 (0.39; 6.06)	3.63 (0.90; 11.92)	6.06 (4.00; 237.00)
mTOR	0.07 (0.01; 0.46)	2.30 (1.00; 653.00)*	2.39 (2.00; 4.10)	2.30 (0.50; 79.00)
70s 6 киназа	0.92 (0.82; 4.00)	0.38 (0.25; 151.86)	0.75 (0.27; 1.17)	2.00 (0.38; 592.00)
4EBP1	0.06 (0.00; 0.74)	0.19 (0.01; 0.25)	2.57 (2.56; 3.32)	2.00 (0.00; 3.80)
PTEN	4.00 (0.24; 6.78)	0.76 (0.50; 61.91)	0.15 (0.02; 1.16)	2.00 (0.76; 642.00)**
Transcription and growth factors				
NFkBp65	0.76 (0.50; 9.56)	51.20 (44.80; 99.60)*	1.16 (0.13; 1.76)	69.66 (51.2; 475.00)**
NFkBp50	23.24 (10.21; 32.00)	0.50 (0.38; 96.86)	1.22 (0.49; 16.00)	8.57 (0.38; 520.00)
VEGFR2	0.12 (0.06; 0.13)	0.76 (0.01;112.64)	0.23 (0.13; 7.26)	6.06 (0.76; 481.00)
VEGF	0.34 (0.13; 0.38)	1.00 (0.00; 12.13)	0.82 (0.03; 1.96)	12.13 (6.06; 21.00)**
CAIX	1.34 (0.50; 2.62)	1.65 (0.19; 4.00)	0.56 (0.08; 0.96)	8.57 (0.19; 211.00)
HIF-2	0.83 (0.25; 1.27)	1.00 (0.43;3.03)	1.50 (0.13; 1.54)	3.03 (0.76; 108.00)
HIF-1	5.35 (0.25;16.62)	1.00 (0.50;246.83)	7.28 (0.13; 13.72)	6.06 (0.50; 481.00)
VHL	2.68 (0.52; 79.25)	150.32 (32.80; 225.9)*	1.70 (0.72; 3.81)	62.76 (32.5; 100.5)**
Hormone reception				
TRIM16	0.07 (0.00; 1.40)	0.18 (0.09; 1.73)	4.55 (0.26; 7.63)	6.80 (0.18; 104.00)
Brn3α	0.65 (0.04;1.54)	0.38 (0.22; 21.59)	3.06 (1.23; 11.59)	8.00 (0.38; 406.00)
AR	0.89 (0.01; 2.16)	0.25 (0.19; 621.15)	0.57 (0.02; 1.44)	2.00 (0.19; 302.00)
ERα	1.02 (0.44; 1.36)	1.52 (1.32; 31.80)*	1.53 (1.07; 8.00)	1.52 (1.00; 16.00)
ERβ	1.00 (0.42; 1.40)	1.00 (0.76; 6.70)	0.52 (0.10; 1.71)	0.78 (0.76; 124.00)

*, the significance of the differences in markers in the tumor tissue; where the status of *BRAF*-V600E is identical in the tumor and metastases compared to non-concordant tissues, p <0.05; **, the significance of the differences in markers in the tissue of metastases, where the status of *BRAF*-V600E is identical in the tumor and metastases in comparison with non-concordant tissues, p <0.05;

## Discussion

Change in PTCs molecular profile followed by the overexpression transcription and growth factors is typical of papillary thyroid cancer (Spirina et al, 2019). The *BRAF*-V600E mutation is a significant unfavorable prognostic sign in the PTC progression, predicting the response to anti-cancer therapy and diseases’ outcome (Stanley et al., 2012). A molecular profile determines the rate of the direction of processes in oncogenesis. Previous studies have shown its association with the transcription and growth factors overexpression (Spirina et al., 2018). Its relationship with the molecular markers expression profile in the primary tumor and the LNMs was noted. For a primary tumor in genetic alteration, we found molecular markers overexpression associated with aggressive tumor growth. On the contrary, for metastatic tissue, a decrease in the RNA level molecular markers and a change in the molecular profile characteristics were noted. The study revealed the contribution of the HIF-1 and VHL in aggressive PTC behavior. We showed VHL significance in PCT oncogenesis, resulting in the LNMs alteration and the angiogenesis switching on. Low VHL expression was associated with the angiogenesis initiation, accompanied by the transcription and growth factors increase (Stanojevic et al., 2014; Todorović et al., 2018).

It should be noted the molecular mechanisms of cancer spreading, metastasis formation, and LNMs affection in PTC are not studied yet. The single data evidence the *BRAF*-V600E point mutation status does not affect the likelihood of regional lymph node involvement (Kurtulmus et al., 2016; Gawin et al., 2019). In turn, it is known the fact the *BRAF*-V600E mutation protective effect. A decrease in the lung damage in PTC, despite the presence of unfavorable clinical parameters that determine a high risk of developing metastases in the lungs (larger tumor size and the presence of metastatic foci (more than 0, 9 cm) and surgical treatment over a year) was found (Lu et al., 2015). 

The hormone receptors and transcription factors play a significant role in PTC progression (Stanley et al., 2012; Spirina et al., 2019). An increase in AR, ERβ mRNA level in the PTCs was revealed against the background of an ERα and ERβ expression imbalance (Spirina et al., 2018). However, no studies have been carried out in the LNMs. Simultaneously, the decrease in TRIM16 expression and ERα detected in metastases with a positive *BRAF*-V600E status. It probably, confirms the previously identified data regarding protection against lung metastases development in PTC (Walts et al., 2014).

The *BRAF* positive status in primary tumors is an unfavorable prognostic factor related to predicting the effect of the targeted therapy and radiotherapy (Califano et al, 2018). It is known the *BRAF*-V600E mutation in LNM may not be related to the invasive characteristics of PTCs (Vuong et al., 2017). As a result of the study, the significance of the concordance in the *BRAF* status was revealed. Both *BRAF* positive and negative status in primary tumors and LNMs led to the low mRNA levels of studied indicators. Simultaneously, in the concordance absence, growth in molecular markers was observed, accompanied by oncogenesis activation. Mutation loss in metastases was found to be a sign of aggressive cancer cell behavior. The revealed fact explains the fact of a PTCs favorable prognosis in most patients, which is associated with the presence of concordance in the *BRAF*-V600E status in the primary tumor and LNMs in more than 90% of patients (Sohn et al., 2016).

In conclusion, the heterogeneity in the primary tissue’s expression profile and the metastases was noted. The *BRAF*-V600E mutation can affect the molecular characteristics both in the primary cancers and LNMs. In tumor tissues, the intensity of oncogenesis is higher in comparison with metastatic ones. In patients with the mutation, an increase in the expression of the studied marker in the primary tumor was shown, in contrast to the metastases, where the molecular factors’ expression was reduced. The molecular factors variability in the primary tumor and LNMs determines the tumor’s progression. The significance of the HIF, VHL expression, and the *BRAF*-V600E point mutation in the PTC’s development was noted. The discrepancy between the mutant status in the primary tumor and metastatic tissues may be an essential factor associated with the cancer spreading.

## Author Contribution Statement

Liudmila Spirina - manuscript drafting; Irina Kovaleva - review-analysis; Svetlana Chizhevskaya – study concept.

## Aknowledgements

### Students thesis approval

Scientific body or any part of it was no approved as a student thesis

### Ethical committee

The Ethical Committee of Cancer Research Institute of Tomsk National Research Medical Center approved the research.

### Conflict of interests

None.
